# Susceptibility of fabrics in office furniture to microbial attack: microbial burden and health implications

**DOI:** 10.1186/2047-2994-4-S1-P36

**Published:** 2015-06-16

**Authors:** LO Egwari, MI Oniha, OO Ayepola

**Affiliations:** 1 Biological Sciences, Covenant University, Ota, Nigeria

## Introduction

Hygiene is advocated as major in the control of infectious diseases; however an area of neglect is furniture we come in contact with daily. Furniture is made of diverse materials; wood, glass, metals, plastics, rubber, clothing fabrics or their combinations. Colonization of furniture made from different types of material and possible health implications are reported.

## Objectives

We investigated the associated health risk in the use of furniture made from different materials.

## Methods

The office setting was used and tables and seats were sampled for presence and burden of microorganisms. Also visual inspection was made for evidence of microbial attack and degradation of fabrics. Degree of susceptibility of different materials in furniture was assessed. Sampling was done after the surface was wiped with dry cloth and after cleaning with detergent solution only and in addition disinfectant.

## Results

Rubber fabrics were more susceptible to colonization while metal surfaces were least. Fungi were the predominant colonizers especially, *Penicillium* and *Aspergillus* species. *Candida* spp were isolated in 14% of the furniture sampled. Bacteria diversity included the Gram positive and Gram negative, but particular were the preponderance of *Pseudomonas*, *Proteus*, and *Klebsiella*. The use of dry cloth to wipe the surface had negligible role in reducing the burden of colonizing organisms. Cleaning with detergent solution reduced microbial burden by 35% while application of disinfectant yielded 90% reduction. Clothing fabric haboured more microorganisms but showed no visual signs of microbial attack.

## Conclusion

Furniture may serve as a pool for breeding pathogenic organisms if not properly maintained. Regular cleaning with detergent and disinfectant is advocated.

## Disclosure of interest

None declared.

